# Impact of Cyclooxygenase Inhibitors in the Women's Health Initiative Hormone Trials: Secondary Analysis of a Randomized Trial

**DOI:** 10.1371/journal.pctr.0010026

**Published:** 2006-09-29

**Authors:** Judith Hsia, JoAnn E Manson, Lewis Kuller, Mary Pettinger, John H Choe, Robert D Langer, Marian Limacher, Albert Oberman, Judith Ockene, Mary Jo O'Sullivan, Jennifer G Robinson, for the Women's Health Initiative Research

**Affiliations:** 1 Department of Medicine, George Washington University, Washington, D. C., United States of America; 2 Division of Preventive Medicine, Brigham and Women's Hospital, Harvard Medical School, Boston, Massachusetts, United States of America; 3 Department of Epidemiology, University of Pittsburgh School of Public Health, Pittsburgh, Pennsylvania, United States of America; 4 Fred Hutchinson Cancer Research Center, Seattle, Washington, United States of America; 5 Department of Medicine, University of Washington, Seattle, Washington, United States of America; 6 Department of Family Medicine, University of California San Diego, La Jolla, California, United States of America; 7 Department of Medicine, University of Florida, Gainesville, Florida, United States of America; 8 Department of Medicine, University of Alabama Birmingham, Birmingham, Alabama, United States of America; 9 Department of Medicine, University of Massachusetts Medical School, Worcester, Massachusetts, United States of America; 10 Department of Obstetrics and Gynecology, University of Miami, Miami, Florida, United States of America; 11 Department of Medicine, University of Iowa, Iowa City, Iowa, United States of America

## Abstract

**Objectives:**

We evaluated the hypothesis that cyclooxygenase (COX) inhibitor use might have counteracted a beneficial effect of postmenopausal hormone therapy, and account for the absence of cardioprotection in the Women's Health Initiative hormone trials. Estrogen increases COX expression, and inhibitors of COX such as nonsteroidal anti-inflammatory agents appear to increase coronary risk, raising the possibility of a clinically important interaction in the trials.

**Design:**

The hormone trials were randomized, double-blind, and placebo-controlled. Use of nonsteroidal anti-inflammatory drugs was assessed at baseline and at years 1, 3, and 6.

**Setting:**

The Women's Health Initiative hormone trials were conducted at 40 clinical sites in the United States.

**Participants:**

The trials enrolled 27,347 postmenopausal women, aged 50–79 y.

**Interventions:**

We randomized 16,608 women with intact uterus to conjugated estrogens 0.625 mg with medroxyprogesterone acetate 2.5 mg daily or to placebo, and 10,739 women with prior hysterectomy to conjugated estrogens 0.625 mg daily or placebo.

**Outcome Measures:**

Myocardial infarction, coronary death, and coronary revascularization were ascertained during 5.6 y of follow-up in the estrogen plus progestin trial and 6.8 y of follow-up in the estrogen alone trial.

**Results:**

Hazard ratios with 95% confidence intervals were calculated from Cox proportional hazard models stratified by COX inhibitor use. The hazard ratio for myocardial infarction/coronary death with estrogen plus progestin was 1.13 (95% confidence interval 0.68–1.89) among non-users of COX inhibitors, and 1.35 (95% confidence interval 0.86–2.10) among continuous users. The hazard ratio with estrogen alone was 0.92 (95% confidence interval 0.57–1.48) among non-users of COX inhibitors, and 1.08 (95% confidence interval 0.69–1.70) among continuous users. In a second analytic approach, hazard ratios were calculated from Cox models that included hormone trial assignment as well as a time-dependent covariate for medication use, and an interaction term. No significant interaction was identified.

**Conclusions:**

Use of COX inhibitors did not significantly affect the Women's Health Initiative hormone trial results.

## Editorial Commentary

**Background:** As part of a set of studies known as the Women's Health Initiative trials, investigators aimed to find out whether providing postmenopausal hormone therapy (estrogen in the case of women who had had a hysterectomy, and estrogen plus progestin for women who had not had a hysterectomy) reduced cardiovascular risk as compared to placebo. Earlier observational studies had suggested this might be the case. The trials found that postmenopausal hormone therapy did not reduce cardiovascular risk in the groups studied. However, there was a concern that medication use outside the trial with nonsteroidal anti-inflammatory drugs (NSAIDs), and specifically the type of NSAID known as COX-2 inhibitors, could have affected the findings. This concern arose because it is known that COX-2 inhibition lowers levels of prostacyclin, a molecule thought to be beneficial to cardiovascular health, whereas estrogen increases prostacyclin levels. Evidence from randomized trials and observational studies has also shown that patients treated with some COX-2 inhibitors are at increased risk of heart attacks and strokes; the cardiovascular safety of other NSAIDs is also the focus of great attention. Therefore, the authors of this paper aimed to do a statistical exploration of the data from the Women's Health Initiative hormone trials, to find out whether NSAID use by participants in the trials could have affected the trials' main findings.

**What this trial shows:** In this reanalysis of the original data from the trials, the investigators found that the effects of hormone therapy on cardiovascular outcomes were similar among users and non-users of NSAIDs, confirming that use of these drugs did not significantly affect the results from the Women's Health Initiative hormone trials.

**Strengths and limitations:** The original hormone trials were large, appropriately randomized studies that enrolled a diverse cohort of participants. Therefore, a large number of cardiovascular events occurred in the groups being compared, allowing this subsequent analysis to be done. One limitation is that use of COX-2 inhibitors in the trial was low; therefore, the investigators were not able to specifically test whether COX-2 inhibitor use (as opposed to NSAID use generally) might have affected their findings.

**Contribution to the evidence:** The investigators did not set out specifically to evaluate the cardiovascular safety of particular medications in this study. Rather, they wanted to see if these NSAIDs could have modified the effects of the hormone therapy. The secondary analysis done here shows that the main findings from the Women's Health Initiative hormone trials were not significantly affected by use of NSAIDs outside the trial.

## INTRODUCTION

The relationship between cyclooxygenase (COX) inhibition and coronary heart disease (CHD) risk is currently the focus of intense scrutiny [1,2]. The putative increase in CHD risk with selective COX-2 inhibitors has been attributed to reduction in atheroprotective prostacyclin I_2_ levels [3]. Estrogen activates COX-2 in female mice through an estrogen-receptor-mediated mechanism, thereby increasing levels of prostacyclin [4]. This observation has raised concern that COX inhibition might counteract a beneficial effect of estrogen on prostacyclin levels and, in fact, account for the absence of cardioprotection with estrogen in recent randomized trials [5].

Mammals have two isoforms of COX. COX-1 is expressed in most tissues and mediates activities such as vascular homeostasis and gastroprotection [6]. COX-2 is induced at sites of inflammation and mediates inflammatory responses [7], making its blockade a target for treatment of arthritis and postoperative pain. Low-dose aspirin inhibits COX-1 [8], traditional nonsteroidal anti-inflammatory drugs (NSAIDs) inhibit COX −1 and COX-2 [9], and selective COX-2 inhibitors such as rofecoxib, celecoxib, and valdecoxib selectively inhibit COX-2 [10].

The Women's Health Initiative hormone trials unexpectedly demonstrated no overall reduction in coronary risk [11], and a suggestion of harm with combination estrogen with progestin [12]. This analysis evaluates the hypothesis that COX inhibition with NSAIDs modulated the effect of postmenopausal hormone therapy on coronary risk in the Women's Health Initiative randomized hormone trials.

## METHODS

**Figure 1:**
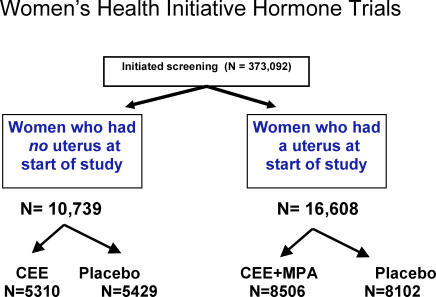
Women's Health Initiative Hormone Trials CEE, conjugated equine estrogens; MPA, medroxyprogesterone acetate.

The design, recruitment, randomization, data collection, intervention, and outcomes ascertainment procedures for the Women's Health Initiative hormone trials, including CONSORT diagrams, have been described in detail elsewhere [13–16]. Also see Figure 1.

### Participants and Interventions

Between November 1993 and October 1998, 16,608 postmenopausal women, aged 50–79 y with intact uterus, were randomized to conjugated estrogens 0.625 mg plus medroxyprogesterone acetate 2.5 mg daily (Prempro; Wyeth Pharmaceuticals, Madison, New Jersey, United States) or placebo in the estrogen plus progestin trial, and 10,739 women with prior hysterectomy were randomized to conjugated estrogens 0.625 mg daily (Premarin; Wyeth Pharmaceuticals) or placebo in the estrogen alone trial (Figure 1). The estrogen plus progestin trial was stopped ahead of schedule after 5.6 y of follow-up upon recommendation of the Data and Safety Monitoring Board because of increased breast cancer risk [16]; the estrogen alone trial was stopped ahead of schedule after 6.8 y of follow-up by the National Institutes of Health because of increased stroke risk and lack of cardioprotection [17].

### Outcomes

#### Medication use.

Participants were asked to bring all medications, including prescription medications, over-the-counter medications, vitamins, minerals, and bulk fiber supplements to clinic for inventory at baseline and at years 1, 3, and 6. Over-the-counter medications taken at least twice a week for the preceding 2 wk, supplements taken at least once a week, and all prescription medications were recorded. Aspirin use indicates a dose of at least 80 mg taken at least twice weekly. NSAIDs and selective COX-2 inhibitors were recorded regardless of dose if they met the frequency of use criteria. Continuous use indicates reported use at baseline and at each follow-up inventory; some use indicates use at some, but not all, medication inventories.

#### Clinical outcomes.

Clinical outcomes were identified from semiannual medical update questionnaires and confirmed by medical record review. CHD death and hospitalized myocardial infarction were confirmed by central adjudicators, the latter using an algorithm that included symptoms, cardiac enzymes, and electrocardiograms [18]. Coronary revascularization was confirmed by centrally trained local adjudicators.

### Statistical Methods

Cox proportional hazard models were stratified by age, prevalent CHD, and randomization in the dietary modification trial [13], and adjusted for coronary revascularization at baseline. The first set of Cox models stratified participants by NSAID use at baseline. The second set of models included a main effect for randomization assignment in the hormone trial and use of aspirin ≥80 mg daily, other NSAIDs, and selective COX-2 inhibitors as time-dependent covariates, and an interaction term. All reported *p-*values are two-sided. Analyses were carried out by the coordinating center statistics unit using the SAS system for Windows version 9 (SAS Institute, Cary, North Carolina, United States).

## RESULTS

Adherence, follow-up, and clinical outcomes in the randomized trials have been previously reported [11,12,16,17].

### Baseline Data

For the individual hormone trials, baseline characteristics were balanced between the active intervention and placebo groups [16,17]. Women with intact uterus in the estrogen plus progestin trial had generally lower prevalence of CHD risk factors than women with prior hysterectomy in the estrogen alone trial (Table 1). For example, the average body mass index of women in the estrogen plus progestin trial was 28.5 ± 5.9 kg/m^2^ compared with 30.1 ± 6.2 kg/m^2^ in the estrogen alone trial. Prevalent hypertension was identified in 36.1% versus 47.7% participants in the two trials, respectively, at baseline, and self-reported diabetes mellitus requiring medication was reported by 4.4% versus 7.7% of participants in the two trials, respectively. The annualized rate percent of myocardial infarction/CHD death was 0.56% for the placebo group in the estrogen alone trial [11], compared with 0.33% for the placebo group of the estrogen plus progestin trial [12].

**Table 1:**
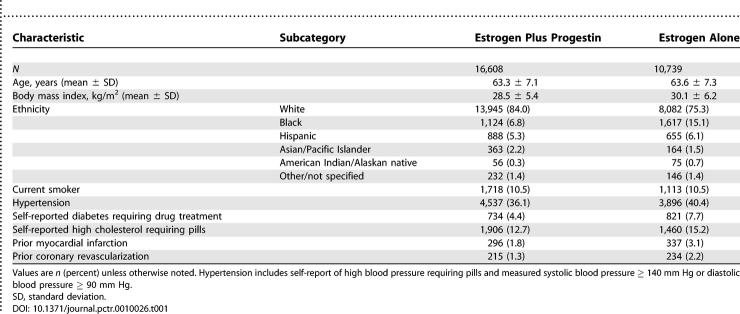
Baseline Characteristics of Hormone Trial Participants

### Outcomes and Estimation

#### COX inhibitor use.

Use of aspirin, traditional NSAIDs, and selective COX-2 inhibitors is shown in Table 2. Among women who used traditional NSAIDs in the estrogen alone trial, 48% and 51% of those assigned to conjugated estrogens and placebo, respectively, used ibuprofen alone (over-the-counter or prescription), 16% and 17%, respectively, used naproxen alone (over-the-counter or prescription), and 31% and 29%, respectively, used other prescription NSAIDs. The remainder took various combinations of ibuprofen, naproxen, and other prescription NSAIDs. Among women taking NSAIDs in the estrogen plus progestin trial, 56% of women in each treatment group took ibuprofen, while 14% of those assigned to estrogen with progestin and 15% of those assigned to placebo took naproxen. In each treatment group, 25% took other prescription NSAIDs.

**Table 2:**
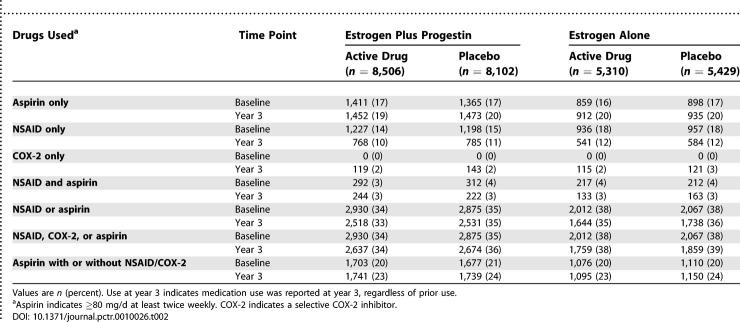
Use of Aspirin, NSAIDs, and Selective COX-2 Inhibitors at Baseline and at Year 3

Celecoxib and rofecoxib were approved by the Food and Drug Administration in 1999, after completion of baseline visits for the hormone trials. Consequently, no women were taking selective COX-2 inhibitors at study entry. During the course of the trial, a small proportion of women began taking these agents.

#### Randomized hormone assignment and COX inhibitor use.

Hazard ratios and 95% confidence intervals are shown for coronary risk with randomized hormone assignment, stratified by NSAID use (Table 3). Among women reporting no NSAID use, the hazard ratio for myocardial infarction/coronary death was 1.13 (95% confidence interval 0.68–1.89) with estrogen plus progestin, and 0.92 (95% confidence interval 0.57–1.48) with unopposed estrogen. Among women taking aspirin and/or other NSAIDs, confidence intervals for CHD risk were similar and spanned unity for both hormone trials. For three strata (none, some, and continuous NSAID use), the *p-*value for interaction with hormone assignment was 0.92 for estrogen with progestin and 0.82 for estrogen alone.

**Table 3:**
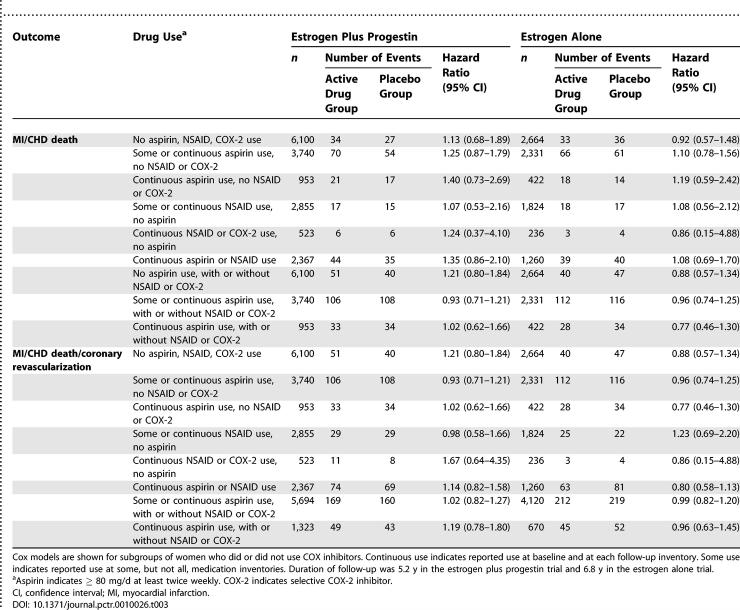
CHD Risk with Postmenopausal Hormone Therapy, Stratified by COX Inhibitor Use

Similarly, for the composite outcome of myocardial infarction/coronary death/coronary revascularization, the hazard ratio among women reporting no NSAID use was 1.21 (95% confidence interval 0.80–1.84) with estrogen plus progestin, and 0.88 (95% confidence interval 0.57–1.34) with unopposed estrogen. For women taking NSAIDs, hazard ratios were similar and 95% confidence intervals for the composite coronary outcome also spanned unity. For three strata of NSAID use (none, some, and continuous), the *p-*value for interaction with hormone assignment was 0.63 for estrogen with progestin and 0.30 for estrogen alone.

A separate set of Cox models evaluating the risk of myocardial infarction/coronary death or myocardial infarction/coronary death/coronary revascularization with NSAID use included a main effect for randomization assignment in the hormone trials, along with NSAID use as a time-dependent covariate and an interaction term (Table 4). No significant interaction was identified between randomization assignment and use of aspirin only, other NSAID only, NSAID with aspirin, or NSAID without aspirin for either CHD outcome.

**Table 4:**
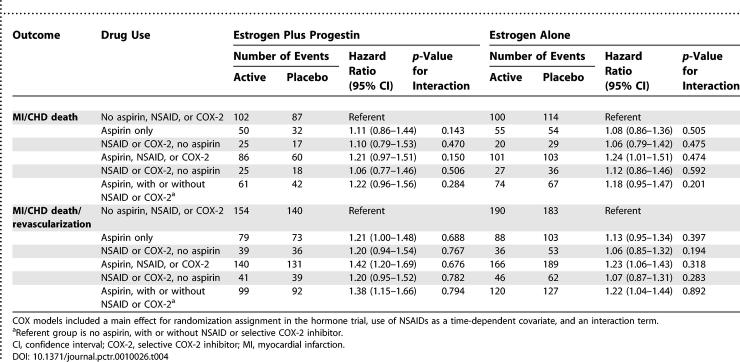
CHD Risk with COX Inhibitor Use: Interaction with Randomized Hormone Assignment

## DISCUSSION

### Interpretation

COX inhibitor use did not significantly modulate the effect of either unopposed conjugated estrogens or combined conjugated estrogens with medroxyprogesterone acetate on coronary risk in the Women's Health Initiative randomized hormone trials. The effects of hormone therapy on risk of coronary events were generally similar among users and non-users of COX inhibitors, and no significant interactions were observed.

The strengths of our study include the systematic ascertainment of clinical coronary outcomes, the large number of CHD events, the randomized, placebo-controlled design, and the periodic re-inventory of medications, permitting inclusion of COX inhibitor use as a time-dependent covariate (Table 4). Limitations include the fact that use of aspirin <80 mg daily was not recorded, that only about 20% of women were using NSAIDs, and that only a few percent used selective COX-2 inhibitors. Thus, we were unable to adequately test the possibilities that concurrent use of very low dose aspirin or exclusive use of selective COX-2 inhibitors might modulate CHD risk among women taking postmenopausal hormone therapy. Further, the numbers of clinical events were small for some categories of COX inhibitor use.

### Generalizability

Characteristics of the Women's Health Initiative hormone trials include the large, diverse cohort and wide geographic distribution of clinical sites. Each trial tested a single regimen: when they were designed, the unopposed estrogen and combination estrogen with progestin regimens were selected because they were the most commonly prescribed regimens in the United States.

Since observational studies of CHD risk with postmenopausal hormone therapy provided misleading results [19], determining the interaction between estrogen use and COX inhibition would necessitate a factorial randomization to estrogen or placebo and to COX inhibitor or placebo in a population at sufficiently high CHD risk. Such a trial is unlikely to be carried out, leaving the exploration of this issue to studies using animal models, which have their own limitations [20].

### Overall Evidence

This analysis is not intended to assess the coronary risk associated with COX inhibitor use. Although we have more complete information about over-the-counter NSAID use and CHD risk characteristics, including physical activity and diet, than some other epidemiologic analyses, this issue is best examined in randomized trials [21–25] because of intrinsic biases in COX inhibitor use related to patient selection and treatment indications.

Iatrogenic imbalance between COX-1 and COX-2 activities has been proposed as a mechanism underlying both favorable and unfavorable cardiovascular effects of drugs [5,26]. COXs synthesize prostacyclin I_2_ and thromboxane A_2_ from arachadonic acid. Prostacyclin I_2_, predominantly a product of COX-2, is a vasodilator that inhibits platelet aggregation and smooth muscle proliferation, effects that might be expected to reduce acute coronary syndromes and stroke. Thromboxane A_2_, produced by COX-1 in platelets, is a vasoconstrictor that stimulates platelet aggregation, an effect that might be expected to increase cardiovascular risk. Selective COX-2 inhibitors reduce prostacyclin without inhibiting production of platelet-COX-1-derived thromboxane A_2_, a pharmacologic effect that has been hypothesized to underlie the putative adverse cardiovascular effects of these agents [24]. In contrast, NSAIDs reduce formation of both prostacyclin and thromboxane A_2_, with individual drugs differing in their relative blockade of COX-1 and COX-2 activities. Naproxen and aspirin predominantly inhibit COX-1, whereas diclofenac, etodolac, and meloxicam predominantly inhibit COX-2 [27]. Participants in the Women's Health Initiative hormone trials consumed a variety of NSAIDs, encompassing a range of ratios of COX-1:COX-2 inhibition.

Estrogen increases expression of COX-2 and production of prostacyclin I_2,_ effects that have been proposed to underlie its apparent cardioprotective effects in animal models [28]. Female low-density lipoprotein cholesterol receptor knockout mice developed more aortic plaque if they also lacked the prostacyclin receptor; this phenomenon was not observed in male mice. The prostacyclin-receptor-deficient female mice also demonstrated increased oxidative stress and platelet activation [4]. In cultured mouse aortic smooth muscle cells, estrogen exposure increased COX-2 expression and prostacyclin formation [4].

In view of these new findings and of the public health impact of the Women's Health Initiative hormone trials, we felt it was important to assess any possible impact of COX inhibitor use on the hormone trial results. Although this analysis cannot conclusively determine whether exogenous estrogen could ever modulate CHD risk with COX inhibition, it does confirm that use of COX inhibitors did not significantly affect the Women's Health Initiative hormone trial results.

## SUPPORTING INFORMATION

CONSORT Checklist Click here for additional data file. (1.6 MB DOC)

Trial Protocol Click here for additional data file. (147 KB PDF)

## Abbreviations

CHD: coronary heart disease
COX: cyclooxygenase
NSAID: nonsteroidal anti-inflammatory drug
