# Supplementary material for: Impact of Cyclooxygenase Inhibitors in the Women's Health Initiative Hormone Trials: Secondary Analysis of a Randomized Trial
Source: PLoS Clin Trials. 2006 Sep 29;1(5):e26. doi: 10.1371/journal.pctr.0010026 (PMC1584256; doi:10.1371/journal.pctr.0010026)
Supplement: Trial Protocol [file pctr.0010026.sd002.pdf]

SECTION 5

HORMONE REPLACEMENT TRIAL

INTRODUCTION

Hormone replacement therapy with estrogen or estrogen and progestin is commonly prescribed for women whose ovarian function is diminishing normally at mid-life or who have had their ovaries removed (oophorectomy). Women receive these exogenous hormones to control menopausal symptoms such as hot flashes, night sweats, or vaginal dryness, or to possibly minimize their risk of heart disease or fractures. It is the effectiveness of hormone replacement in preventing heart disease and fractures that the Hormone Replacement Therapy (HRT) component of the Clinical Trial (CT) seeks to investigate.

Women participating in the HRT component of the CT will be randomized in a double-blinded fashion based on the presence or absence of a uterus.

- Women with a uterus will be randomized to one of two arms:
  - a) Conjugated equine estrogen (CEE) 0.625 mg per day + medroxyprogesterone (MPA) 2.5 mg per day continuously
  - b) Placebo estrogen + placebo progesterone
- Women without a uterus will be randomized to one of two arms:
  - a) Conjugated equine estrogen (CEE) 0.625 mg per day
  - b) Placebo estrogen

Note: WHI will use formulations containing the CEE currently marketed as Premarin™ and the MPA currently marketed as Cycrin™.

Guidelines and requirements for implementation of the HRT component of the CT are included in this section. In addition, a *Consulting Gynecologist Handbook* containing HRT-related clinical procedures and guidelines, is provided in *Vol 2, Appendix H*. Each CC receives one copy of this *Appendix H* in a separate binder for use by the CC consulting gynecologists. Each Clinical Center (CC) may devise policies and schedules for themselves within these recommendations and requirements to produce optimal performance in the Women's Health Initiative (WHI) and quality care for its participants.

Clinical Center staffing for implementation of the HRT may also vary. Completion of physical measurements, interviewing, and follow-up tasks may be done by appropriately trained and WHI-certified medical technicians, medical assistants, interviewers, or RNs. Clinical tasks such as clinical breast exams, pelvic exams, and endometrial aspirations (EA), however, will require staff with the appropriate state licensure and supervision. (e.g., physician, nurse practitioner, physician assistant, registered nurse, or licensed practical nurse.) In addition to local *clinical* standards of care (not other research protocols), WHI certification is required for consistent data collection.

5.1 HRT Eligibility Issues

To be eligible for the HRT, women must meet the eligibility criteria for the CT as a whole. *Vol. 1 - Study Protocol and Policies, Section 1 - Protocol, Section 4.4 - Study Population* lists the additional criteria that a woman must meet to be eligible for the HRT. Most of these additional criteria are for safety reasons. It is the philosophy of WHI to avoid the administration of hormone replacement therapy to women who could be harmed by it. Each item of eligibility must be checked carefully before a woman is randomized into the HRT.

5.1.1 Informed Consent

A woman must sign the HRT consent form before you perform any clinical trial-specific activities. See *Section 4.2.4.9 - CT Informed Consent* for procedures for reviewing the HRT Consent Form with the woman.

5.1.2 Eligibility Based on Baseline Gynecologic Evaluations

5.1.2.1 Exclusions Based on Baseline Clinical Breast Exam Findings.

All baseline breast exams must be done by appropriately licensed (for clinical practice), supervised (if required), and certified WHI staff.

Women with findings that suggest possible malignancy on baseline breast examination will be temporarily ineligible for HRT until cleared by correlation with mammogram and/or ultrasound findings or clinical judgment and documentation. Tissue diagnosis will be necessary in some cases to establish a clear non-malignant status. Suspicious findings include masses that are new or changed or nipple discharge. Participants with these findings should be evaluated by the CC consulting gynecologist or their primary physician. If cancer is excluded, the woman is eligible.

Note that these exclusions are for all CT participants, including those interested only in DM. A normal mammogram report (or a follow-up to an abnormal mammogram that excludes malignancy) is a requirement before you can randomize a woman into the CT and for the woman to continue in the HRT component. (See *Vol. 2, Section 12 - Mammography*.)

5.1.2.2 Exclusions Based on Baseline Pelvic Exam Findings

All baseline pelvic exams must be done by appropriately licensed (for clinical practice) and certified WHI staff.

Women with findings that suggest possible malignancy on baseline pelvic examination will be temporarily ineligible for HRT until cleared. Vulvar, perineal, and vaginal findings that suggest possible malignancy include external lesions, ulcerations, or growths. Bimanual exam findings include uterine enlargement of greater than 12-week size or adnexal enlargement or masses. Participants with these findings should be evaluated by the CC consulting gynecologist or their primary physician, if they prefer. If cancer is excluded, the woman is eligible.

5.1.2.3 Exclusions Based on Baseline Pap Smear

All women interested in HRT must have a baseline Pap smear (endocervical smear in women with an intact cervix, vaginal cuff in women without a cervix). A Pap smear performed during the 12 months before SV2 will be accepted as baseline, if the cytology report can be obtained or if a verbal report from the performing physician’s office is obtained.

Note that a woman who has had a subtotal hysterectomy may still have a cervix, and therefore should be followed regularly at years 3, 6, 9 and closeout.

The following classifications and actions are to be followed for the baseline Pap smear:

Classification	Eligibility Status	Further Action
Cancer (Invasive only)	Ineligible	Refer urgently to primary physician.
High-grade SIL (moderate dysplasia, severe dysplasia, carcinoma in-situ [CIS])	Temporarily ineligible	Refer to primary physician for evaluation. If cancer is excluded, woman is again eligible.
Low-grade SIL (mild dysplasia, atypical squamous cells, human papilloma virus)	Eligible	Inform primary physician of Pap smear results
ASCUS / AGCUS (Atypical squamous or glandular cells, undetermined significance)	Eligible	Inform primary physician of Pap smear result

Often for low-grade SIL or ASCUS, a second smear in 3 to 6 months would be recommended; some gynecologists do immediate colposcopy. These follow-up procedures to an abnormal Pap smear should not be done by CC staff or physicians, but should be referred to the participant's health care provider.

Note: CCs may decide to adopt more conservative guidelines than these.

In cases where the Pap report reads either "Insufficient material" or "No endocervical cells," the following guidelines should be used:

- If the Pap is from the vaginal vault, or if the woman has had a Pap smear at least once in the past and has not had dysplasia, this Pap result will be considered satisfactory and the participant will be eligible.
- If the woman has never had a Pap smear or has had dysplasia in the past, a repeat smear should be taken at the 6-month visit, but she will still be eligible.

5.1.2.4 Exclusions Based On Baseline Endometrial Evaluation

All women with a uterus interested in the HRT must have an endometrial evaluation at baseline that is classified as normal. Histological assessment is the preferred method of evaluation. Women may not *choose* to have a transvaginal uterine ultrasound instead of an endometrial biopsy. An endometrial aspiration or diagnostic D&C performed during the 12 months before SV2 will be accepted at baseline if pathology results can be obtained. A normal classification for the WHI baseline aspiration includes the following diagnoses by the local pathologist:

- No endometrial tissue identified
- Insufficient specimen
- Normal atrophic endometrium
- Normal secretory endometrium
- Normal proliferative endometrium

If endometrial fluid (quantity sufficient to fill the biopsy cannula, about 1.5 cc) is encountered at the baseline biopsy, and subsequent histology is benign, obtain a transvaginal ultrasound examination for the purposes of measuring the endometrial stripe and assessing the ovaries. If the total stripe width (exclusive of fluid) is $\leq$ 5 mm and there are no pelvic abnormalities, the woman is considered eligible for HRT.

If the pathology report reveals an endometrial polyp, and no other excluding diagnosis is present, no further evaluation of the endometrium is required for eligibility.

An abnormal classification for the WHI baseline endometrial aspiration is based on the following diagnoses by the local pathologist:

- Simple hyperplasia present
- Complex (adenomatous) hyperplasia present
- Atypia present carcinoma in-situ [CIS])
- Neoplasm, a cancer, present

Note: Women with any of the above diagnoses on baseline endometrial aspiration will be ineligible for the HRT. If the biopsy is investigated and found not to be endometrial cancer, the participant may be eligible for DM only. *Figure 5.1 – Baseline Endometrial Aspiration Actions* outlines actions to be taken based on the baseline aspiration. Note that the baseline endometrial aspiration will not have a central pathological review.

5.1.2.5 Exclusions Based on Baseline Transvaginal Uterine Ultrasound

If an endometrial aspiration is unsuccessful due cervical stenosis a second attempt should be made unless it is determined by the CC consulting gynecologist that it is unlikely to succeed. In that case, a transvaginal uterine ultrasound should be performed. An endometrial thickness ≤ 5 mm on the transvaginal uterine ultrasound will be accepted as normal and therefore the participant would be eligible for the HRT. If the endometrial thickness is greater than 5 mm, make a referral to her primary care provider. The participant may undergo a diagnostic D&C for increased endometrial thickness, and if the pathology report shows a normal result (see *Section 5.1.2.4 – Exclusions Based On Baseline Endometrial Evaluation*), the participant may be eligible. Transvaginal ultrasound measurement of the endometrial thickness may not be accurate if the uterus has either intramural or fibroids. Therefore, when the stripe cannot be measured uniformly due to this (or any other) problem, ultrasound only will not be considered acceptable as a baseline measure. If the baseline test is an ultrasound (unable to enter the uterus for endometrial sampling), and significant fluid is reported, the woman is considered eligible if the total stripe width (exclusive of fluid) is ≤ 5 mm and the pelvic structures are normal. If the transvaginal report reveals an endometrial polyp and no other excluding diagnosis is present, no further evaluation of the endometrium is required for eligibility. If neither the baseline endometrial aspiration nor the transvaginal ultrasound can be performed because of participant discomfort, inability to enter the vagina, or any other reason, the participant is ineligible for HRT.

Figure 5.1
Baseline Endometrial Aspiration Actions

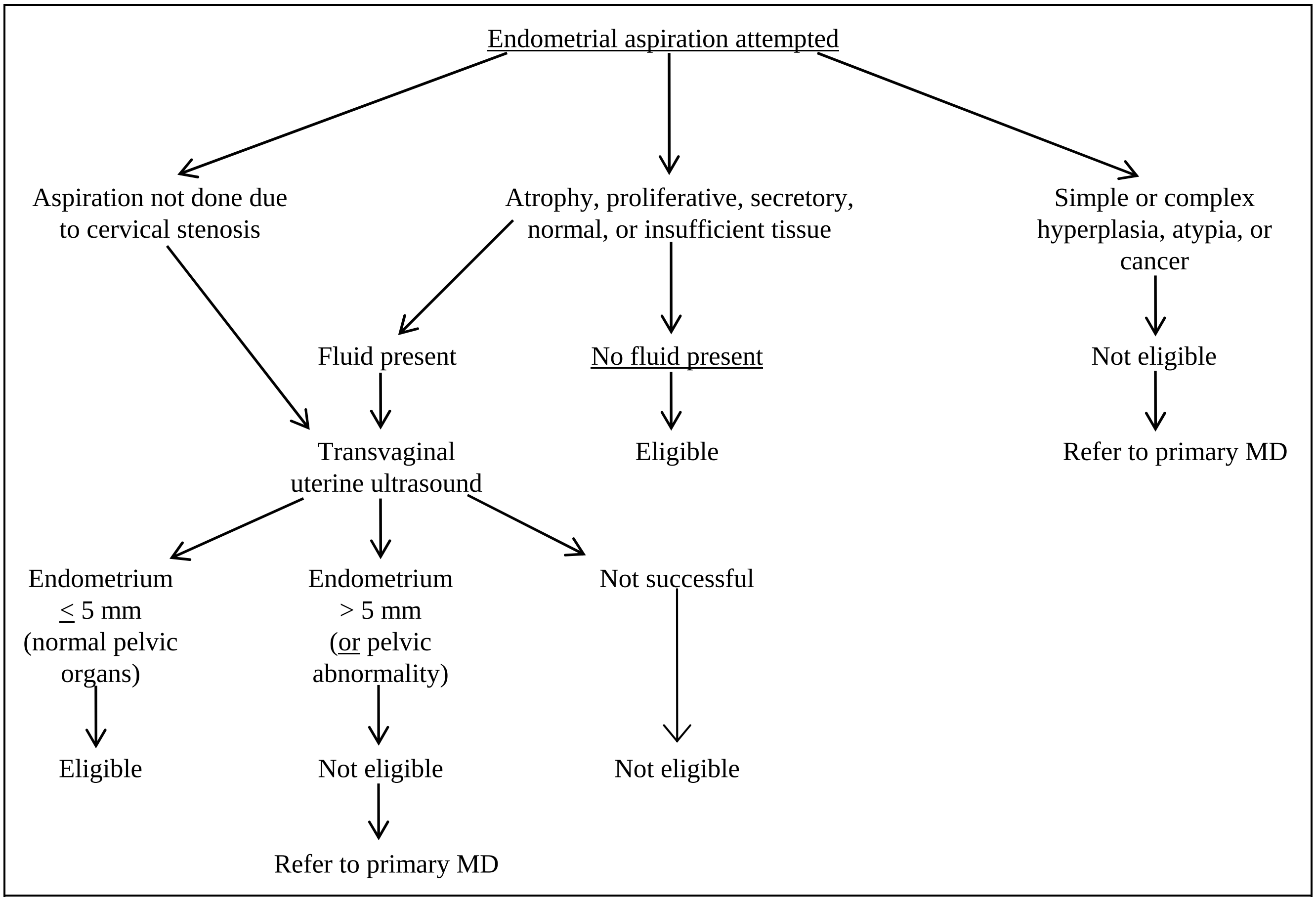

5.1.3 HRT ”Washout” Period for Screenees Already on HRT

Some women who are interested in participating in the HRT may be currently taking hormone replacement therapy. Women must be off hormone replacement therapy for three months before Screening Visit 1 (SV1) before they can be considered for participation in HRT. Therefore, women must washout of prescription estrogen or progesterone in oral, patch, injectable, or vaginal delivery (cream or ring) form, and oral and injectable testosterone (testosterone cream does not require a washout). Women who use herbal estrogen preparations should be asked to discontinue them, but a washout is not required. The hormone washout is to give women time to adjust to any symptoms they may have once they stop taking hormones. Some women will have severe persistent menopausal symptoms off hormone replacement therapy and are ineligible for the HRT because they would not be able to tolerate being randomized to placebo.

Women who report currently taking hormones during the initial screening interview will be instructed to see their primary physician or gynecologist if they are considering participation in the HRT. A letter from the CC explaining WHI, its goals, and the purpose of the washout may also be sent to the primary physician or gynecologist at this time (see *Figure E.1.4 - Model Letter to HRT Participant Health Care Provider*). They should discontinue their hormone replacement therapy only under the advice and guidance of their physician. Most women will need to be tapered off hormones by their physician to control withdrawal symptoms.

See *Section 4.5.4.1 - Tasks to be Repeated Post-HRT Washout* for women who initiate hormone washout after starting the screening process.

5.2 Initiating the HRT Intervention

The HRT intervention consists of the study pills, the *HRT Handbook*, *Form 53 - HRT Calendar*, and discussions with participants on the specifics of the intervention. (See *Appendix F, Figure F.3.2 - HRT Handbook*.) Use the four steps below when providing the study enrollment pills and the Handbook to each participant at the end of SV1 or beginning of SV2. Review them as necessary at subsequent contacts.

5.2.1 Step 1 - Introducing the HRT Intervention to the Participant

The following guide contains specific information on beginning the HRT intervention. Refer also to *Section 17.2.3 - Reasons for Poor Retention and/or Adherence* and *Section 17.2.4 - Strategies for Retention Challenges* for additional suggestions on helping a participant stay on study medications.

5.2.1.1 Providing the *HRT Handbook*

Welcome the participant to the HRT and congratulate her on her participation so far. Hand her a copy of the *HRT Handbook*. Explain that this handbook contains the basic information she will need and that you and she will cover some of that information right now. Tell her that if she ever has any questions she should ask them as you go along so that she will not forget them.

5.2.1.2 Reviewing the Importance of the HRT

Refer to the *HRT Handbook*. Review the importance of the HRT to women's health and to the field of prevention science. Remind the participant of her importance to the study and of her generosity in volunteering her time and effort. Discuss the scientific outcomes of the study (e.g., effects of HRT on heart disease and osteoporosis, who should take hormones, etc.) and why it is so critical to learn about cardiovascular disease in women. Also reassure the participant by emphasizing the many safety points in the HRT study. Use appropriate sections of the *HRT Handbook.*

5.2.2 Step 2 - Taking the HRT Study Pills

5.2.2.1 Instructions for Taking Pills

Give the participant her bottle of enrollment pills. Explain the label contents. Review the instructions for taking the HRT study pills as described in the *HRT Handbook*. These include what to take, when and how to take the pills, and how to store the pills. Include the fact that there are no special requirements about taking the pill with food, beverages, etc. Discuss what to do if taking the pills is difficult (e.g., swallowing). Ask the participant if she wants a non-child resistant cap. (If she does, see *Section 15.1.3 - Child Resistant Caps* for procedures for giving her one.)

5.2.2.2 Using the Pill Organizer

Give the participant a seven-day pill organizer and show her what it is for and ways to use it to remind her to take pills. Discuss using it for other pills she must take and remind her that the HRT study pill storage rules apply to pills in the organizer as well as pills in the bottles.

5.2.2.3 Designing a Reminder System

Ask the participant how she is going to remember to take the pill every day. Ask her about other pills or daily activities she has and how she remembers to do these things. Help her to integrate the HRT study pill with other daily pills or activities. If she (or you) seems concerned with her ability to remember, or if she indicates that she has had trouble in the past remembering to take pills or perform other daily activities, help her to design a cueing or reminder system to help her remember. Explain what a cue is (a cue is anything that will remind the participant to take her pill every day). Consider time of day, placement of cue or reminder, ease of seeing the cue, consistency of cue, etc., when designing the reminder system. Ask the participant how she will

remember to take her study pills when she is in an unusual situation (e.g., on vacation, traveling, weekends, etc.) Use appropriate sections of the *HRT Handbook*.

5.2.2.4 Identifying and Building Skills

Ask the participant if she thinks she will be comfortable performing all of the activities needed to participate in the HRT study. Clarify and discuss her areas of concern. Recommend specific behavioral goals and identify steps to achieve those goals. For example, remembering to take pills daily could include cueing the participant for a specific time and place to take the pill, having a supply of pills to take at that time and place, being assertive in order to take the pill in the presence of others, etc. Rehearse these new behaviors with her. Ask her to let you know how they worked at the next contact.

5.2.2.5 Review

Review all the steps involved in taking HRT study pills with the participant. Ask the participant questions that allow you to be sure she understands all the information and is ready to participate. An example of such questions include, ”How are you going to remember to take your pills?” Remind her to call with any questions.

5.2.3 Step 3 - Understanding Symptoms

5.2.3.1 Identify Fears and Beliefs

Ask the participant what she is feeling about starting on the pills. Identify any fears, worries, or apprehensions that she may have about taking study pills. Ask her if she, her friends, or family members have had negative experiences with hormones. Ask her what she expects to happen to her and what the consequences of these events will be. Listen and acknowledge the participant’s fears and beliefs about HRT, even if you feel they are unfounded. Correct any misperceptions she may have about the potential effects of HRT.

5.2.3.2 Reviewing Possible Symptoms

Discuss with the participant possible symptoms that she may experience. Indicate that each woman is different and that her experience may be the same or different from other women. Discuss her study pill to date, if appropriate. Emphasize the long-term nature of the program, including long-term gains and the fact that symptoms should be reduced by the end of the first year. Let her know that experiencing symptoms is normal and usually diminishes with time. Discuss with her the ways that the trial procedures promote participant safety and remind her that if she has any questions she should call the CC right away. Use appropriate sections of the *HRT Handbook*.

5.2.3.3 Review

Review the issues she brought up about her previous experiences and fears of HRT. Also, identify any issues about which you have more up-to-date information to allay her fears and concerns in the future.

5.2.4 Step 4 - Discussion of Routine Monitoring

5.2.4.1 Reasons for Monitoring

Explain to the participant the reasons for monitoring (e.g., ensuring the safety of the participant and collecting follow-up data).

5.2.4.2 Self-Monitoring

Show the participant with a uterus *Form 53 - HRT Calendar* and instruct her in its completion. Stress the importance of her regular completion of this form during the first year to track any bleeding she may have. Tell

her this form will also help to remind her to take her daily pills and that you will review this form together at her next CC visit.

5.2.4.3 Symptom Monitoring

Discuss the ways in which symptoms of HRT participants are monitored regularly. The current methods include *Form 53 - HRT Calendar*, *Form 10 - HRT Management and Safety Interview*, and many of the other questionnaires that each participant completes regularly. Indicate that these data will allow us to monitor the safety and progress of each participant in the HRT.

5.2.4.4 Clinical Monitoring

Discuss the various clinical monitoring activities that will occur throughout the trial, including gynecological exams. Discuss the endometrial aspiration with the participant. Inform her that a small number of participants will be asked to have an endometrial aspiration at follow-up visits. Refer to the *HRT Handbook*. Remind her that by doing these clinical monitoring activities, we will be able to better monitor safety and progress of the HRT.

5.2.4.5 Review

Review the basic schedule of follow-up and monitoring that occurs as part of the HRT. Ask the participant questions to determine if she understands her responsibilities and activities in the trial and the reasons for them. Ask her if she has any other questions at this time.

5.2.5 Randomization Visit

The randomization visit (SV3) is an important intermediate step in the HRT intervention. *Section 4.6.3.5 - Remote Site Randomizations* provides requirements and options for conducting remote site randomizations with HRT participants. Use the following three steps to monitor adherence and any symptoms during the enrollment period.

5.2.5.1 Step 1 - Weighing Pills

Ask for the participant's pill bottle at the beginning of the visit. Use the scale to weigh the remaining pills and record the pill weight in WHILMA using the procedures in *Section 15.6.22. - Bottle Weighing Procedures* and *Vol. 5 - Data System, Section 7.3.3.5 - Medication Adherence Collection*. Record the adherence rate on *Form 10 - HRT Management and Safety Interview* to use during the visit. Note that WHILMA does not take altered dose regimens into account. Do not weigh the pills in front of the participant or tell her that you are doing so.

5.2.5.2 Step 2 - Monitoring Adherence and Symptoms

At the randomization visit, women in HRT will participate in a short interview (*Form 10 - HRT Management and Safety Interview*). During this interview they will be asked about adherence patterns and strategies they may have used. This interview will continue to build rapport between participants and CC staff and ensure that worrisome symptoms are identified for each participant. (See the instructions for *Form 10- HRT Management and Safety Interview* for questions to be asked during this interview.)

Administer the interview using the following steps:

- Check the hysterectomy status of the participant in WHILMA by calling up the participant status screen or looking at a recent visit plan for the participant or asking the participant.
- Inform the participant that you would like to ask her a series of questions to help you keep track of her health.
- Administer *Form 10 - HRT Management and Safety Interview*.

- Review *Form 53* for report of bleeding.
- Refer the participant to the Clinic Practitioner as appropriate.

It is critical that you attend to symptoms a participant may describe at this point. She may report symptoms that are not due to study pills, but can be treated using the suggestions in *Section 5.4.1 – Minor Symptoms*.

5.2.5.3 Step 3 - Reviewing Key Points

Review the key topics of the HRT intervention at the randomization visit. Use the materials in *Section 5.2 – Initiating the HRT Intervention* steps 1-4 above, to guide the discussion. Key points include:

- Introduction and use of the *HRT Handbook*.
- Taking the study pills.
- Understanding symptoms.
- Routine monitoring.

5.3 Follow-Up Contacts with HRT Participants

5.3.1 Identifying Problems at Participant Contacts

Use every contact with a participant to identify issues or problems with regular pill taking or related symptoms. Use the materials in *Section 5.2 – Initiating the HRT Intervention*, Steps 1-4 above, to guide the discussion. Review *Section 15.8 - Managing Adherence* and *17.2.5 - Intensive Adherence Program* and develop strategies to improve pill taking behavior.

5.3.2 SERMs Handout

CCs are not required to give the handout *WHI Update – What You Should Know About SERMs* to participants. However, if CCs decide to give participants information on SERMS, they must use this handout. (See *Appendix F – Required CC Printed Materials*, *Figure F.3.10*.)

5.4 Managing Symptoms

Trial participants may experience adverse effects related to study interventions. These may range from mild inconvenience to life-threatening illnesses, or possibly death. Take great care to identify potential adverse experiences early so that appropriate and prompt treatment, referral, and study medication stoppage decisions can be made. Each of the CT components will have different expected adverse effects. Most of the adverse effects will be seen in HRT participants.

5.4.1 Minor Symptoms

5.4.1.1 Educate the Participant About Possible Symptoms

Several adverse effects are common among women taking hormone preparations, but other symptoms may be related to changes with aging or menopause. Not all women will have such symptoms, and the severity and frequency of symptoms will vary among women, as will the responsiveness of symptoms to hormone dose or regimen changes. When the participant starts on HRT study pills, inform her, by reviewing the HRT Handbook, of the various possible minor adverse effects that could occur. Tell her that these symptoms are in most cases not harmful, but that she should contact the CC if any of the symptoms become very uncomfortable or severe. Reassure her that the minor symptoms associated with HRT study pill use usually resolve spontaneously within 6-12 months of starting the medications.

5.4.1.2 Participants' Reporting of Minor Symptoms

Participants may report symptoms to CC staff in several different ways:

- Unscheduled or scheduled phone calls to the CC.
- Reporting symptoms to CC staff at the 6-week, semi-annual, or annual *HRT Management and Safety Interview* (*Form 10*).
- Recording in the "Notes" section of *Form 53 - HRT Calendar*.
- Responding to questionnaire items.

5.4.1.3 Initial Management of Minor Symptoms

The initial management of minor symptoms consists of palliative treatment, with the goal of keeping the participant in the original treatment arm. Many of these recommendations are included in the *HRT Handbook* (see *Appendix F, Figure F.3.2* for model) provided to the participant. Try these palliative treatments for a minimum of one month before initiating the dose step-down algorithm. Many women with HRT symptoms will be able to tolerate them with reassurance and simple relief measures.

If the participant experiences symptoms during the enrollment period, suggest the palliative measures described below. If the participant still experiences severe symptoms that she finds intolerable, complete *Form 11 - Consent Status* and mark that she declined further screening.

Discuss each minor symptom with the participant. The following steps may be used as guidelines:

- Ask about frequency, duration, and intensity, and record it on the "Notes" section of *Form 10 - HRT Management and Safety Interview*.
- Ask how much it interferes with daily activities.
- Give advice based on the guidelines below.
- Tell the participant that these symptoms are not health-threatening, but that she can call the CC should any of the symptoms become very uncomfortable or severe.
- Reassure her that these symptoms usually decrease after 6-12 months.

- Tell her that a CC staff member will call her back in one month to ask if she is still having any problems. Use a reminder system (tickler file or computer report) to track the calls.

WHI Clinic Practitioners (CPs) will not write prescriptions for treatment of symptoms, but may recommend over-the-counter medications, except aspirin. Vitamins should NOT be routinely recommended by CC staff for menopausal symptoms.

- Headaches: Recommend over-the-counter analgesics; i.e., acetaminophen (650 mg every 4-6 hours) or ibuprofen (200-400 mg every 4-6 hours). Before recommending ibuprofen or other non-steroidal anti-inflammatory drugs, be sure to ask the participant if any doctor ever told her not to take these medications or if she suffers from peptic ulcer disease or kidney failure. Do not recommend aspirin. If she has persistent or severe headaches, refer her back to her primary physician.
- Fluid retention, bloating, or change in bowel habits: Recommend dietary salt restriction, increased fluid intake, avoidance of caffeine (coffee, tea, chocolate and caffeinated cola beverage), and increased dietary or supplemental fiber intake. Do not prescribe diuretics. If a participant has fluid retention severe enough to warrant diuretic therapy, refer her to her primary physician. Such severe fluid retention will be a basis for review and, if persistent, for dose reduction or stopping the HRT.
- Irritability, depression: Determine sleeping and eating patterns. If symptoms are thought to be due to study medication, consider palliative measures (caffeine reduction, reduced salt intake, increased fluids, mild exercise and increased social activity). If these are ineffective, use the dose reduction algorithms described in *Section 5.4.1.4 – Step-Down Dose Management for Refractory Symptoms.* If the participant seems seriously depressed or if she or a family member is worried about her mood, she should be evaluated by a CP urgently. If you suspect that the participant has a depressive illness, refer her to her primary physician. If the CP judges the participant to be a danger to herself or others, make the appropriate emergency referrals. The following are suggested depression screening questions for the CP:

  “In the past two weeks or more, did you feel sad, blue, or depressed or lose pleasure in things you usually cared about or enjoyed?”

  If yes: “Have you felt sad or depressed much of the time in the past year, even if you felt okay sometimes?”

  ”In the past two weeks or more have you thought that life isn’t worth living?”
- Breast tenderness or swelling: Recommend caffeine (coffee, tea, chocolate) restriction and well-fitting, supportive bras. Recommend reduced salt intake and increased fluid intake. Reassure the participant that these symptoms usually resolve within two to three months.
- Sleep disturbances: Recommend mild exercise earlier in the day, caffeine and alcohol restriction, relaxing activity before bedtime, and avoiding heavy meals in the late evening. If you suspect a depressive illness, the participant must be evaluated by a CP. (See *Irritability, anxiety, depression* above).
- Nausea: Recommend that HRT study pills be taken in the evening or with food, to minimize problems with nausea. Consider referring the participant to her primary physician for further evaluation if the nausea is persistent, severe, or accompanied by vomiting.
- Vaginal discharge: Counsel the participant when starting study pills to expect some increase in vaginal discharge. However, the presence of foul odor, itching, dysuria, irritation or burning could be evidence of infection. These women should be referred to their primary physician for evaluation and treatment. Some women with a persistent irritating discharge may have atrophic vaginitis, which is usually treated with hormone replacement therapy. Recommend to the participants and their physicians that a non-estrogen vaginal lubricant (e.g., Replens, Astro-Glide, Lubron, K-Y jelly) should be tried initially. If the symptoms remain worrisome, the participant’s physician will be asked to consult with the CC consulting gynecologist. If they (the Consulting GYN and the participant’s provider) agree that local tissue stimulation by estrogen (delivery by cream or ring system) is indicated, the weekly dosage should be consistent with the published prescribing guidelines (e.g., PDR or patient package insert). Women who

are given vaginal estrogen cream by their physicians will remain blinded and on their assigned study drugs. Include the participant's use of vaginal estrogen products when you do the current medications update at the next annual visit.

- Increased skin pigmentation: Inspect any reported changes in skin color, and if warranted, refer the participant to her primary care provider.
- Muscle or joint pain: Recommend acetaminophen or an over-the-counter non-steroidal anti-inflammatory agent (if no contraindication exists). Do not recommend aspirin. If a participant has significant joint pain, swelling, or erythema, refer her to her primary physician.

5.4.1.4 Step-Down Dose Management for Refractory Symptoms

Figure 5.2 – Hormone Step-Down Management for Symptoms describes the step-down procedures to follow if a participant does not respond to the palliative treatments suggested above. Participants' dosage may be decreased to improve symptoms. After a month's trial, attempts should be made to advance to the original daily dose or the highest tolerable dose. Warn the participant that some spotting or bleeding might occur with the step-down, but should eventually decrease and/or stop.

Call the participant one month after reducing her HRT study pills to determine her response to the initial palliative management of her symptom(s). Document responses in the participant's file. If she reports continued symptoms, refer her to the CP.

You must complete a *Form 54 - Change of Medications* each time you add, change, stop, or alter the HRT study or open-label pills or dosage. Do not complete a *Form 54* for changes initiated by the participant that you have not agreed to.

1) Tell the participant to decrease her HRT study pill regimen to one tablet for five days of the week (e.g., Monday through Friday) and to continue in this manner for one month. Record the initiation of step-down in the participant's contact notes and on *Form 54 - Change of Medications*. Warn the participant that this change in pill schedule may cause the appearance of, or increase in existing vaginal bleeding. In one month, the CP should call to ascertain her response to the step-down dosing.
2) If the participant tolerates the altered dosing, gradually advance her back to the daily schedule: six days/week for two weeks; then seven days/week. If this is not tolerated, further step-down to every other day dosing may be started. The CP should call the participant in one month to ascertain her response to medication dose. If she does not tolerate the return to the full dose, tell her to return to the highest dosing schedule she can tolerate. Complete *Form 54 - Change of Medications* to reflect each new dosage.
3) If steps 1 and 2 are not tolerated, the CP should contact the CC's consulting gynecologist. Present the participant's symptoms and step-down response. The gynecologist will decide on treatment changes based on the participant's treatment arm. The CC consulting gynecologist can contact the CC Unblinding Officer who can execute a database function for unblinding and tell only the consulting gynecologist the participant's randomized arm. See *Vol. 5 - Data System, Section 6.5 - Unblinding Procedures* for a full description of duties of the CC Unblinding Officer.

   In rare cases, the CC consulting gynecologist will decide to change the participant's study pills or discontinue HRT altogether for refractory minor symptoms. Such decisions must be made in concert with the CC PI or their designee, who will not be made aware of the participant's identity during a decision about changing or stopping study pills.

4) If unblinding reveals to the consulting gynecologist that the participant is on active hormone, the CC consulting gynecologist (who is unblinded) and participant (who is *not* unblinded) must decide whether the symptoms are tolerable and she can continue her study pills, or if the participant cannot tolerate the symptoms and should discontinue her pills. If the participant is on placebo, the CC consulting

gynecologist should refer her to her primary care provider, as her symptoms may reflect an underlying medical problem.

5) If unblinding reveals to the consulting gynecologist that the participant is on active hormone, the consulting gynecologist can change the participant's hormone regimen to manage these symptoms. (See *Figure 5.2 – Hormone Step-Down Management for Symptoms*.)

6) If the participant with a uterus tolerates a lower dose of MPA, then attempt to gradually advance her back to a daily schedule (PERT every day). If a participant cannot be completely advanced to PERT, the minimal amount of MPA 2.5 mg allowable is 3 pills/week.

7) If the participant with a uterus cannot tolerate any MPA, consider discontinuing her study pills.

For any referral back to the primary physician, a CC physician or CP will call the primary physician, send a letter or facsimile information, and describe the study to the physician. If the primary physician insists on stopping study pills, ask him/her to accept a step-down dosage first, and in either case to maintain the integrity of the double-blind randomization. The philosophy of WHI is that the CCs are not responsible for the primary health care of the participants. The CC physicians and practitioners will work in every possible way to maintain and foster each participant's relationship with her own source of primary medical care and to assist the primary physician with that individual's care. However, the CC will also make every attempt to avoid compromising the trial protocol, unless absolutely necessary for the safety of a participant.

5.4.2 Management of Menopausal Symptoms

Rarely, some women may continue to have postmenopausal symptoms after being randomized to study pills. This will be uncommon in WHI as all women who were previously receiving HRT will have been asked to washout of their previous HRT for three months before SV1. Those women who experienced severe postmenopausal symptoms after these three months were not eligible for the HRT component.

Management of continued postmenopausal symptoms will be done blinded. *Figure 5.3 - Continued Menopausal Symptoms Management* outlines the steps to be taken for management of postmenopausal symptoms on study medications:

1) Women complaining of intolerable hot flashes or night sweats will use palliative treatments (e.g., wearing cool, light clothing and avoiding stressful situations) for one month.

2) If their symptoms persist, increase their study pills to two pills per day for one month.

3) If she continues to have symptoms, consider stopping her study pills and referring her to her primary physician, as these symptoms could indicate other medical problems.

4) If the symptoms decrease, continue the two pills per day for two more months.

5) If the symptoms continue, decrease the regimen as necessary to one pill a day. If this is not tolerated, discontinue the study pills and refer the participant to her primary physician for evaluation.

6) Record each of the changes in study pill regimen on *Form 54 - Change of Medications*. If the participant stops using study pills altogether, a *Form 7 - Participant Status* should be completed.

7) Do not recommend vitamin or herbal therapy to alleviate menopausal symptoms.

Figure 5.2
Hormone Step-Down Management for Symptoms

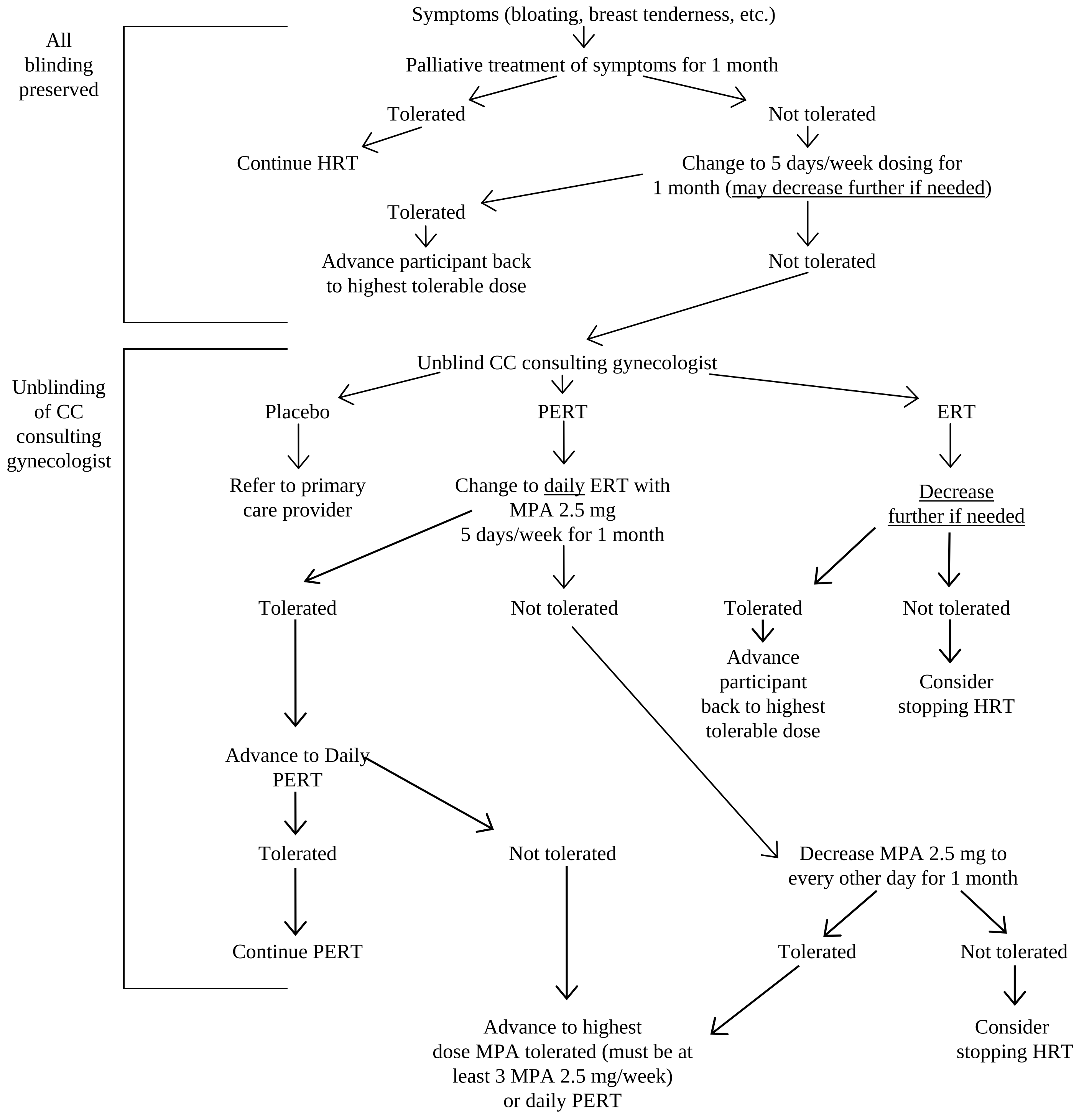

Figure 5.3
Continued Menopausal Symptoms Management

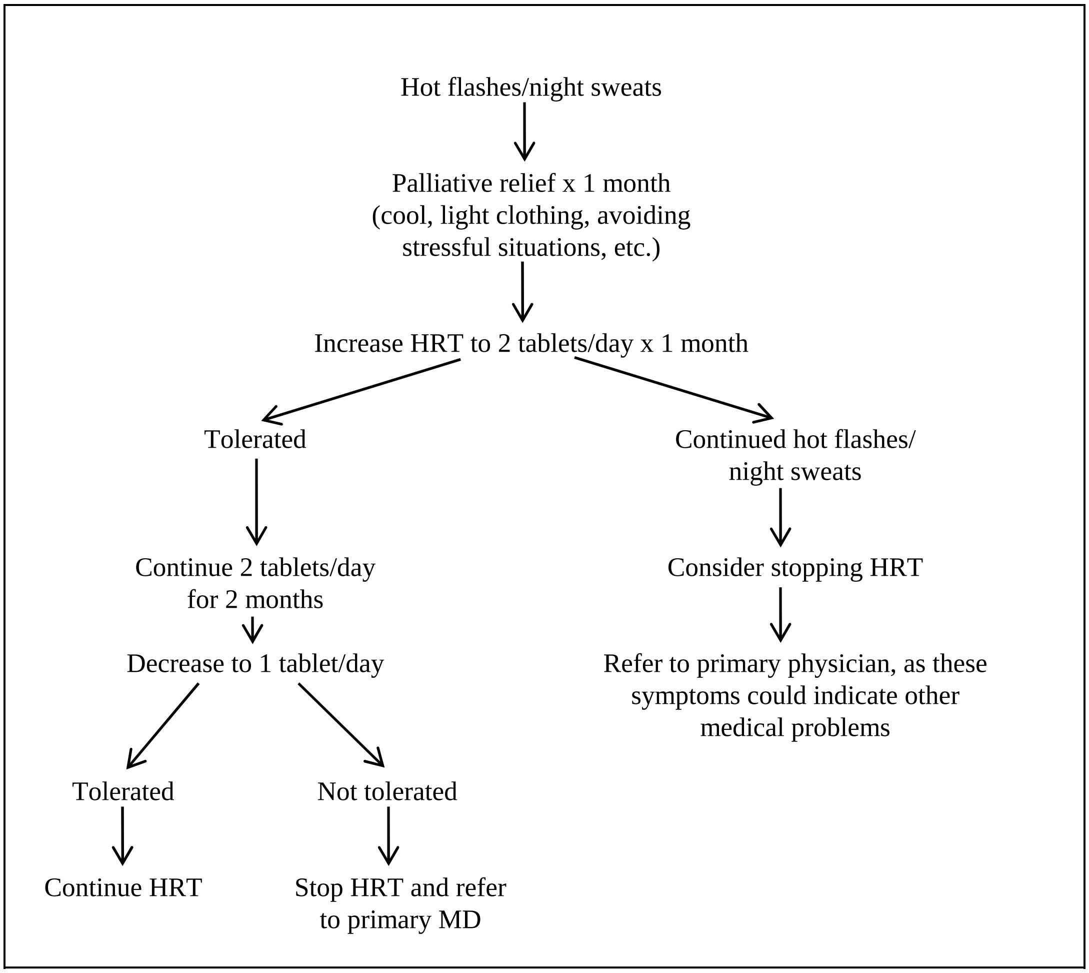

5.4.3 Management of Vaginal Bleeding

E-plus-P participants who present or report vaginal bleeding over 8 weeks from stopping their study pills require further investigation and work-up. CCs should perform an EA on these participants or the participants may have the EA and/or any further work-up with her outside provider. Any abnormal findings will require follow up and management by an outside provider.

Many women participating in the HRT who have not had a hysterectomy will experience vaginal bleeding at some time during the study. For women in the PERT arm, some amount of spotting is to be expected. However, the spotting usually begins to resolve by six to twelve months after starting HRT. Bleeding anytime after 6 months post-randomization into HRT will always need investigation to rule out endometrial disease. In HRT, only the CC consulting gynecologist will, if necessary, be unblinded to the study arm. This will aid greatly in making decisions about initial management of vaginal bleeding.

The management of vaginal bleeding will depend on (a) severity of bleeding, (b) time since randomization, (c) study arm assignment, and (d) endometrial histology. Consideration should also be given to how the endometrium was evaluated at baseline. See *Section 5.4.3.1 – When to Biopsy*.

Inform each woman who has not undergone hysterectomy that bleeding may occur after she starts the HRT study pills. She should call the CC if she has an episode of severe bleeding (bleeding heavier than her regular period used to be or eight or more pads/day). Have the participant speak to the CP, who will review the participant's file and ascertain the amount of bleeding, time since starting study pills, and history of previous bleeding. This CP will complete a non-routine *Form 10 - HRT Management and Safety Interview* (if contact occurs outside of a routine contact) and present the case to the CC consulting gynecologist, who will have

access to the participant's file to determine the results, if any, of previous bleeding work-ups. The case presentation to the CC consulting

gynecologist will include the amount of bleeding, time since starting study pills, and history of bleeding since randomization.

Once a decision for treatment has been made, the participant will be called and either: (1) given a CC appointment for further evaluation during the same week, or (2) reassured and told when to return to the CC.

Participants who have not undergone hysterectomy will be asked to keep a *Form 53 - HRT Calendar* that they will bring with them on each CC visit during the first year post-randomization. The CP will review these calendars when the participant comes to CC visits before scanning. For evaluation of bleeding after the first year, CPs may choose to use *Form 53 - HRT Calendar* to monitor amount, pattern, and number of days of bleeding. However, this form does not need to be entered into the database after the first year.

If a participant has undergone a hysterectomy and reports vaginal bleeding, refer her to her primary care provider as soon as possible. If a participant who previously reported having a hysterectomy is found to have a uterus or uterine tissue, contact your CCC Data Coordinator Liaison before dispensing any HRT study pills. Present the case to the CC consulting gynecologist, who will decide if an endometrial biopsy is necessary. Manage the participant accordingly. (See *Section 9.9.2 – Performing the Pelvic Exam and Pap Smear.)*

5.4.3.1 When to Biopsy

The following procedures are established to avoid unblinding whenever possible, minimize the amount of staff unblinding, and avoid excessive aspirations. Given that most women have had an endometrial evaluation at baseline, bleeding equal to or lighter than a regular period (including moderate amounts of bleeding) during the first six months post-randomization will be assessed and managed by the CP without unblinding of the CC consulting gynecologist.

If bleeding warrants an EA and the participant refuses the exam, a TVUS is acceptable (the participant should not be offered a TVUS unless she refuses an EA). If she refuses both procedures, stop HRT study pills. HRT study pills may be restarted if she has either an EA or TVUS procedure and any abnormal findings are cleared.

- **Bleeding During the First Six Months Post-Randomization –**

  Women whose **baseline endometrial evaluation was by ultrasound** (and not a biopsy), and who bleed beyond three months after starting HRT should have an endometrial biopsy attempted, as biopsy provides a more definitive assessment of endometrial tissue.

  If the participant used a previous D&C or biopsy from an outside primary care provider, which could be more than six months old, ***more immediate evaluation may be necessary***.

  Heavy bleeding (greater than usual menstrual bleeding, or eight or more pads/day) during the first six months post-randomization requires that the CC consulting gynecologist be **unblinded** and direct management or refer to the participant’s primary care provider as appropriate.

  - If the participant is in the **placebo** group, the CC consulting gynecologist should order an endometrial biopsy.
  - If the participant is in the **PERT** group and more than six months have elapsed since the last endometrial assessment (randomization was based on a previous D&C or biopsy from an outside primary care provider) a repeat biopsy will be obtained.

  Participants with **spotting or light to moderate** bleeding during the first six months post-randomization should be assured that this bleeding is expected and reassessed at the next scheduled semi-annual or annual visit.

- **Bleeding After the First Six Months Post-Randomization –**

  The CC gynecologist should be unblinded for all bleeding after six months.

  Heavy bleeding (greater than usual menstrual bleeding, or eight or more pads/day) after the first six months post-randomization requires endometrial biopsy. This bleeding may be due to fibroids or polyps.

Significant uterine findings should be referred to the participant's primary care provider for further evaluation and management.

Spotting or light to moderate bleeding should be presented to the CC consulting gynecologist who decides whether an unscheduled biopsy is necessary based on information from *Form 53 - HRT Calendar* (if available), endometrial evaluation record, and/or treatment arm.

– If the participant is in the **placebo** group, the CC consulting gynecologist should order an endometrial biopsy.

– If the participant is in the **PERT** group, had a normal baseline endometrial evaluation, and:

- **bleeding is decreasing or staying the same, the participant should continue study pills and return for evaluation at next scheduled semi-annual or annual visit;**
- **bleeding is increasing,** biopsy should be done.

5.4.3.2 Management According to Endometrial Histology

All results of diagnostic and subsample endometrial aspirations will be referred to the CC consulting gynecologist who will use the following guidelines to direct further management. Blinding of CC personnel, except for the CC consulting gynecologist, should be preserved.

CPs should keep in mind, however, that they should attempt to return the participant to the standard WHI dose regimen to keep altered dose regimens to a minimum.

<u>**PLACEBO Treatment Arm**</u>

- **Aspiration Not Done Due to Cervical Stenosis**

  CC consulting gynecologist will order an ultrasound evaluation.

  - If the endometrial stripe is less than or equal to 5mm, the participant will continue placebo therapy.

  - If the endometrial stripe is greater than 5mm, the participant should be referred to her primary care provider for further evaluation.

- **Normal, Atrophic, Proliferative, Secretory, or Insufficient Tissue**

  If the biopsy was done routinely, no unblinding or changes need to be made.

  If the biopsy was done non-routinely for bleeding, the participant may continue on her study pills. If bleeding persists, refer the participant back to her primary care provider.

- **Hyperplastic, Atypical, or Cancerous Results**

  If the participant has histologic results of simple or complex hyperplasia, atypia or cancer, discontinue all HRT study pills and refer her to her primary care provider. Send a copy of the pathology report to the participant's primary care provider.

  If a participant with hyperplasia <u>without atypia</u> is treated by her primary care provider, and her EA reverts to normal, she can re-enter the study.

<u>**PERT Treatment Arm**</u>

- **Normal, Atrophic, Proliferative, Secretory, or Insufficient Results**

  If the biopsy was done routinely, no unblinding or changes need to be made.

  If the biopsy was done non-routinely for bleeding, the participant should be reassured and continued for another three months on her study pills and then re-evaluated.

If the participant is bothered by the bleeding, the CC consulting gynecologist has the option of adding medication to attempt to decrease troublesome bleeding.

- For those biopsies showing an **atrophic** result, the CC consulting gynecologist can order an additional 0.3 mg of CEE daily for up to three months per year.

  Some participants who are put on additional CEE 0.3 mg daily will resume bleeding after the CEE dosage is reduced back to the standard regimen of CEE 0.625 mg a day (+ MPA 2.5 mg). For these participants, indefinite use of additional CEE 0.3 mg daily may be considered (*see Section 5.4.3.5 – Indefinite Use of Additional CEE 0.3 mg Daily*). Yearly endometrial surveillance will be required in these cases.

- For those biopsies showing a normal endometrial assessment within one year, the CC consulting gynecologist may administer a course of MPA 2.5-10 mg daily for three months.

  After this 3-month course, the CC consulting gynecologist will re-assess the amount of bleeding. If the MPA treatment led to decreased bleeding, the CC consulting gynecologist may prescribe another 3-month course of MPA for a total of six months of treatment.

  If after six months the bleeding is still decreasing, try decreasing the MPA dosage. Up to 5 mg of MPA daily may be continued indefinitely with re-evaluation every 6 months.

- For those biopsies showing a normal endometrial assessment within one year and continued bleeding unresponsive to other interventions, CCs may dispense open-label 5 or 10 mg MPA pills to HRT participants as "cyclic MPA" under specific circumstances (see below). This cyclic dosing should be used only as a "rescue therapy" for those HRT participants who have experienced persistent bleeding for at least 6 months and are asking to quit their study pills. Participants may also be placed on a cyclic regimen, so that bleeding patterns are predictable. Refer *to Section 5.4.3.6 – Use Of Cyclic CEE/MPA for HRT Participants* for guidelines for this regimen.

- **Simple and Complex Hyperplastic Results**

  Participants must be unblinded. Complete a *Form 54 – Change of Medications* for each change in study pills.

  - **Simple Hyperplasia**

    The participant may continue **PERT.**

    The CC consulting gynecologist will order MPA 20 mg/day for 3-6 months and repeat the biopsy.

  - **Complex Hyperplasia**

    The participant must stop **PERT.**

    The CC consulting gynecologist will order MPA 20 mg/day for 3-6 months and then repeat the biopsy.

  - **If repeat biopsy results in a report of no change in hyperplasia:**

    After the first 3-6 months of MPA therapy (and appropriate repeat biopsy, as above) - Increase MPA to 30 mg/day for another 3-6 months and re-biopsy.

    After a full 6-12 months of MPA therapy (and appropriate repeat biopsy, as above) - Stop PERT (if not already stopped) and refer to primary care provider.

  - **If repeat biopsy results return to normal after 3-6 months of MPA treatment:**

    Previous diagnosis of simple hyperplasia – Stop additional MPA, continue PERT.

    Previous diagnosis of complex hyperplasia – Re-institute PERT with additional MPA 2.5 mg/day for the remainder of the study. Re-evaluate every 6 months.

  - **If repeat biopsy results in a report of conversion from complex to simple hyperplasia:**

After the first 3-6 months of MPA therapy (and appropriate repeat biopsy, as above) – Treat with MPA 30 mg for 3-6 months and re-biopsy.

After a full 6-12 months of MPA therapy (and appropriate repeat biopsy, as above) - Treat with MPA 20 mg for another 3-6 months and re-biopsy.

– **If biopsy results in a report of <u>progression</u> of hyperplasia:**

CC consulting gynecologist should stop intervention and refer the participant back to her primary care provider who should decide if additional diagnostic procedures are warranted.

If a participant with hyperplasia without atypia is treated by her primary care provider and her EA reverts to normal, she can resume study pills.

Some women may develop an atrophic endometrium and bleed before the 3-6 months of MPA treatment are over. In these cases, if the participant has completed at least 2 weeks of MPA, it would be appropriate to decrease the MPA to 10 mg daily, and re-biopsy within a month. Treatment goals should include using a sufficient course of MPA to convert the endometrium to normal and avoid multiple re-biopsies.

- **Atypia or Cancer**

  Discontinue study pills of participants with a histologic diagnosis of atypia or cancer and refer to their primary care provider. Send a copy of the pathology report to the participant's primary care provider.

5.4.3.3 Management According to TVUS Results

If an endometrial aspiration cannot be obtained or is refused, a TVUS is acceptable. If the TVUS results identify an endometrial stripe > 5mm, refer to the consulting gynecologist. Study pills may be continued if abnormal TVUS findings (stripe > 5mm) are cleared within 6 weeks. A subsequent normal EA or TVUS will clear an abnormal TVUS. Study pills must be discontinued if abnormal TVUS findings are <u>not</u> cleared within 6 weeks. Pills may be restarted once the abnormal findings are cleared.

5.4.3.4 Additional Guidelines for Managing Bleeding after First Year of HRT Study Pills

It is estimated that 20 to 30% of the participants will continue to bleed past the first 6 months to 1 year of taking HRT study pills. The CC consulting gynecologist is unblinded for all. Note that no open-label hormonal treatment should be initiated on participants in the placebo group. Options for management as described for bleeding after the first six months are described below:

- Add CEE 0.3 mg daily for a three-month period. Indefinite use may be considered if the guidelines in *Section 5.4.3.5 – Indefinite Use of Additional CEE 0.3 mg Daily* are followed. This regimen may help those participants who are spotting or bleeding due to an atrophic endometrium.

- Add MPA up to 10 mg daily for up to six months if the participant has been unblinded and has had a normal endometrial aspiration in the past 12 months. CPs may give lower doses of MPA for less than six months based on their clinical judgment.

- Participants may need long term daily MPA 2.5 - 5 mg to stop bleeding. If necessary, this may be started at any time during the study and continued indefinitely. However, this regimen should be assessed at each 6-month contact to attempt to return the participant to standard study regimen.

- Use cyclic regimen if bleeding persists and participant threatens to leave study. (Refer to *Section 5.4.3.6 – Use of Cyclic CEE/MPA for HRT Participants*).

- If a participant experiences heavy bleeding and does not respond to hormone treatment, then consider other possible causes of bleeding, such as polyps or fibroids, and make a referral to the participant's primary care provider, if appropriate.

<u>All medication changes made by the CC</u> should be recorded on *Form 54 - Change of Medications*, including return to standard regimen.

5.4.3.5 Indefinite Use of Additional CEE 0.3 mg Daily.

Some participants who are put on additional CEE 0.3 mg daily will resume bleeding after the CEE dosage is reduced back to the standard regimen of only CEE 0.625 mg a day (+ MPA 2.5 mg). For these participants, indefinite use of CEE 0.3 mg daily can be considered if either:

- The vaginal bleeding restarts after the three-month treatment period (of additional CEE 0.3 mg) is completed; **or**
- The participant indicates that she will discontinue her study pills if she is not allowed to continue on the extra CEE.

Although the expected risk of hyperplasia should be low, the CP caring for participants who continue on this regimen for more than 3 months should <u>either</u>:

- Increase the dose of MPA by adding open-label MPA 2.5 mg for a total dose of 5 mg daily; or
- Perform an endometrial aspiration if extra MPA is not used and the participant continues on the additional CEE for 9 additional months. An aspiration should be repeated on a yearly basis as long as the participant remains on this additional CEE regimen.

The decision to continue the additional CEE must be reviewed by the CC consulting gynecologist on a yearly basis.

5.4.3.6 Use of Cyclic CEE/MPA for HRT Participants

CCs may dispense open-label 5 or 10 mg MPA pills to HRT participants as "cyclic MPA" under specific circumstances (see below). This cyclic dosing should be used only as a "rescue therapy" for those HRT participants with uteri who have experienced persistent bleeding for at least 6 months and are asking to stop their study pills.

a. Identify Participants for the Cyclic CEE/MPA Regimen.

All of the following conditions must be met before CCs may offer an HRT participant a cyclic CEE/MPA regimen:

- The CC consulting gynecologist has been unblinded, knows the participant is on active PERT, and has evaluated and approved this course of action; *and*
- The participant is experiencing persistent (greater than six months), unpredictable vaginal bleeding and is willing to continue the intervention if the bleeding could be predictable; *and*
- Vaginal bleeding has not responded to the use of additional MPA and/or CEE as indicated in the HRT procedures in *Vol. 2, Section 5.4.3.4 – Additional Guidelines for Managing Bleeding After First Year of HRT Study Pills*; and
- The participant has asked to stop HRT study pills because of the bleeding.

b. Cyclic CEE/MPA Regimen

The cyclic regimen to be used is open-label CEE 0.625 mg every day with open-label MPA, 5 or 10 mg, added for the first or last 12-14 days of the month. The CC consulting gynecologist may decide on the exact dosage and duration of MPA based on an evaluation of the individual participant.

c. Implement the Cyclic CEE/MPA Regimen

Use the procedure below to select, dispense, and monitor the use of cyclic CEE/MPA:

- Discuss the participant's case with your CC consulting gynecologist, who will make the decision about implementing a cyclic regimen.
- Discuss with the participant the goal of a cyclic regimen (i.e., to help make the bleeding more predictable) and the administration schedule for this regimen. Remind her that bleeding, much like menstrual cycle bleeding, should occur 1-2 days after finishing the MPA for that month. Counsel her that this regimen does not mean she will stop bleeding entirely. If she agrees to try this regimen, continue with the procedures below.
- Collect her remaining HRT study pills and bottles and do an adherence collection in WHILMA on her remaining HRT study pills.
- Do not unblind the participant to her original treatment assignment. You may explain to her that only the CC consulting gynecologist knows her treatment arm and feels that this change will be a safe way to make her bleeding more predictable.
- Dispense only a six-month supply of the open label pills at this time: 2 bottles of open label CEE 0.625 mg and one bottle of MPA 5 or 10 mg. If her next routine visit is scheduled less than six months form the date of dispensation, collect the dispensed bottles at that routine visit and dispense a new six-month supply.
- Remind the participant when she should take her MPA by marking the designated dates of MPA (progestin) doses for the next six months on the participant's WHI monthly calendar pocket planner (annual incentive item). Emphasize that she will be taking two different pills on certain days of the month (specify days according to the CC consulting gynecologist's decision).
- Ask the participant to complete a *Form 53 – HRT Calendar* for at least the first year after starting cyclic therapy. This will help both her and the CP track vaginal bleeding pattern. (This information does not need to be data entered.)
- Provide the participant with appropriate pill-taking instructions. Tell the participant the actual dates that she should take the medication and that there will be pills left in the bottle. She will need to return the pills and the bottle at her next visit.
- Contact the participant after her first cycle (approximately 34-40 days after the open label pills are dispensed) to assess how she is doing. You may complete a *Form 10 – HRT Management and Safety Interview* at this time.
- Schedule or confirm her next follow-up contact at the appropriate time (semi-annual or annual). Consider contacting her at regular intervals in addition to routine contacts in the future as needed.
- Assess the need to continue the cyclic regimen at every routine contact. The decision to continue cyclic therapy or to resume the standard study pill regimen is left to the discretion of the local CP or CC consulting gynecologist.
- Complete a *Form 54 – Change of Medications*.

5.5 Problems That May Affect Continuation of Study Pills

Major adverse effects associated with HRT are those that are potentially detrimental to the participant. Depending on the severity of the effects, the CC consulting gynecologist may elect to stop study pills temporarily or to stop them permanently (only if absolutely necessary). Very few incidents are expected to be so severe that stopping the study pills will be necessary in either the short or the longer term. Major adverse effects will be communicated to the CC in various ways:

- Participant reports condition or event to CC staff at the 6-week HRT follow-up phone call.
- Condition or event is reported on *Form 33 – Medical History Update*.
- Participant reports condition or event to CC staff at a regularly scheduled semi-annual or annual contact.
- Participant reports condition or event at a non-routine CC visit.
- Participant, family members, or primary care provider calls the CC.

Some major adverse effects will have been resolved by the primary care provider by the time the CC is alerted. In such a situation, the study pills may have already been discontinued (or not stopped at all), particularly if the primary care provider has not been fully aware of the trial requirements. In these cases, because the participant has not been unblinded, the participant, with her primary care provider's agreement, may be restarted on HRT if the condition does not contraindicate use of hormones by trial protocol (or the participant may need to stop her study pills). See also *Section 5.5.6 – Guidelines for Restarting a HRT Participant Who Discontinued Pills for 12 Months or More* and *Section 5.5.7 – Guidelines for Restarting HRT Study Pills for a Participant Who Has Had a MI or Stroke.*

Educate the participant on the conditions that do **require** stopping study pills (see *Section 5.5.3 – Health Problems That Require Temporary Discontinuation* and *Section 5.5.4 – Health Problems Requiring HRT Termination)* and stress the importance of contacting the WHI clinic at the time they occur to ensure safety issues are addressed and to minimize scenarios as the one described above. Complete *Form 7 – Participation Status*, as appropriate. Distribute the HRT Handbook annually to all HRT participants.

5.5.1 Management of HRT After Hysterectomy for Non-Cancerous Condition

Some women in the trial will undergo a hysterectomy for reasons other than cancer, such as persistent bleeding from hyperplasia or fibroids. If a woman is to undergo a hysterectomy, she should inform the CC beforehand. Obtain the reason for hysterectomy from the primary care provider. Hysterectomy status will also be reviewed with participants on *Form 10 - HRT Management and Safety Interview* at each contact.

If the reason is not for cancer, restart study pills when the primary care provider feels that it is safe. All women who undergo a hysterectomy should be issued a new bottle of study pills. This will also ensure that the participant is on the correct study pill for her new hysterectomy status. This change will not require unblinding. Clinical Center staff *must call the CCC Data Coordinator for identification of the new bottle.*

Note that women, who undergo an endometrial ablation but keep their uterus, may continue in their original study arm.

5.5.2 Health Problems Requiring Serious Review

The following should be considered as major adverse effects that will prompt a *serious review* of the need to temporarily or completely stop study pills. (Please consult with your PI and/or Consulting Gynecologist):

- **Symptomatic or active gall bladder disease:** Presence of right upper quadrant pain, nausea, anorexia, or jaundice; liver enzyme abnormalities; or ultrasound-diagnosed cholelithiasis. If a participant has had a cholecystectomy, study pills do not need to be stopped. The decision about continuing study pills should be based on clinical discretion with input from the participant's health care provider.

- **Acute pancreatitis**: Presence of upper abdominal pain, nausea, vomiting, sweating, tachycardia, cyanosis, or elevated serum amylase or lipase.
- **Any hospitalization**
- **Surgery requiring short-term prophylactic anticoagulant therapy** (anticoagulants being used to treat thrombophlebitic events require HRT termination).
- **Hysterectomy for a non-cancerous condition** (See *Section 5.5.1 – Management of HRT after a Hysterectomy for a Non-Cancerous Condition).*

If it is necessary to temporarily stop study pills, follow procedure in *Section 5.5.3 – Health Problems That Require Temporary Discontinuation.*

5.5.3 Health Problems That Require Temporary Discontinuation

The following adverse experiences will result in the temporary discontinuation of the hormone replacement therapy:

- Myocardial infarction
- Stroke
- Surgery involving the use of general or spinal anesthesia. (If a participant is scheduled for surgery, she should be advised to speak with her surgeon to see if she should stop study pills ahead of time.)
- Any illness or injury that results in immobilization requiring strict bed rest for more than one week
- Any severe illness in which HRT is temporarily inappropriate (including newly diagnosed TIAs, retinal vascular thrombosis, or other cardiovascular conditions that may increase a participant's risk for a thrombotic event).

If a participant needs to temporarily discontinue her study pills, complete *Form 54 - Change of Medications* to indicate she has stopped. You do not need to collect her bottle until the next regularly scheduled visit. Maintain contact with the participant and her attending primary care provider to determine if and when she can return to her study medicines. When she is able to restart her pills, she can continue using the same bottle. Complete *Form 54* again to indicate she has restarted. Follow procedures in *Section 5.5.1 – Management of HRT After Hysterectomy for Non-Cancerous Condition* if the participant has a hysterectomy. Depending on the length of time she is off study pills, a *Form 7 - Participation Status* may need to be completed. Refer to *Section 16 - Follow-Up*.

5.5.4 Health Problems Requiring HRT Termination

The following adverse experiences will require permanently stopping HRT medications and recording that action on *Form 7 - Participation Status*. For each of these adverse conditions, documentation will be required before the final decision to permanently stop the medication is made. Documentation can include pathology reports, reports of other diagnostic or surgical procedures, or primary care provider or hospital records.

(Note: deleting meningioma from this list was approved by the Steering Committee in June 2000.)

- Deep vein thrombosis (DVT)
- Pulmonary embolus (PE)
- Starting anticoagulant medications for thrombophlebitic events.
- Endometrial hyperplasia with atypia (see *Section 5.1.2.4 – Exclusions Based On Baseline Endometrial Evaluation.*).

- Starting on estrogen, progesterone, testosterone, or Tamoxifen or other SERMS (those preparations that act systemically, such as pills, shots, transdermal patches and skin implants). If a participant subsequently discontinues use of such preparations, you may restart her on HRT study pills. HRT participants may continue to take WHI HRT study pills while they are using prescribed estrogen, progesterone, or testosterone creams; estrogen suppositories (e.g., VagiFem™); Estring™ (a vaginal estrogen delivery system); or over-the-counter (e.g., herbal) hormone preparations.
- Malignant melanoma
- Endometrial cancer
- Breast cancer including intraductal carcinoma on biopsy or carcinoma in-situ
- Triglycerides above 1,000 mg/dl

Note that participants who require long term corticosteroid therapy after randomization may continue their study pills. However, CPs should encourage participants to discuss additional treatment for prevention of osteoporosis with their own primary care providers.

For endometrial biopsy findings, discontinuation of study treatment should be as follows:

- If findings of simple or complex (adenomatous) hyperplasia persist after appropriate management as outlined in *Section 5.4.3.2 – Management According to Endometrial Histology*, discontinue the medications permanently (complete *Form 7 – Participation Status*). Refer the participant to her primary care provider for evaluation and treatment and mail or fax copies of all pertinent reports to them.
- If a participant has a hysterectomy as part of her treatment, see *Section 5.5.1 – Management of HRT After Hysterectomy for Non-Cancerous Condition* for the necessary steps to restart her study pills. (Complete *Form 7* or *Form 54 – Change in Medications* (as appropriate) again to indicate she is restarting the intervention and dispense a new bottle of study pills.)
- If pathologic findings (on either local or central pathology reading) are atypia or endometrial cancer, permanently discontinue HRT medications, complete *Form 7 - Participation Status*, and refer the participant to her primary care provider for evaluation and management. Notification of the participant and her primary care provider should be done by the CC physician or CP. Mail or fax copies of all pertinent reports to the primary care provider.

For pelvic exam and Pap smear findings, see *Volume 2, Section 9.9.2 – Performing the Pelvic Exam and Pap Smear* and *Section 9.9.3 – Pelvic Exam and Pap Smear Findings*.

5.5.5 Other Issues Requiring Discontinuation of HRT Study Pills

In the event that a participant refuses required routine safety examinations and/or diagnostic follow-up exams for suspicious findings, study pills may need to be discontinued (either temporarily or permanently).

Participants are required to have safety exams completed at specific intervals to stay on study pills. These tasks include CBE, pelvic, Pap smear, and *Form 10* completion. See *Table 16.2- Follow-up Clinical Examinations/Minimum Requirements for HRT Participants* for detailed instruction.

Study pills may be restarted if the participant has the routine exam and/or recommended diagnostic follow-up exams and any abnormal findings are cleared. Complete *Form 54 – Change of Medications* when stopping or resuming study pills.

5.5.6 Guidelines for Restarting a HRT Participant Who Discontinued Pills for 12 Months or More

In the event that a participant chooses to restart her HRT study pills after discontinuing her pills for 12 months or more (and it is appropriate to do so), the following procedures are required to be completed and reviewed before study pills can be dispensed.

- Mammogram (*Form 85*) **must be completed within last 12 months.**

- Clinical Breast Exam (*Form 84*) **must be completed within last 12 months.**
- Pelvic Exam (*Form 81*) **must be completed within last 12 months.**
- Pap Smear (*Form 92)* **must be completed within last 3 years.**
- *HRT Management and Safety Interview (Form 10)* **must be completed at the time of the restart.**

All examinations must be considered normal. An endometrial aspiration is **not required** to restart a participant unless there was abnormal bleeding since the intervention was stopped. It is also advised to seek approval and support of the participant's PCP if her intervention was stopped primarily for medical reasons.

5.5.7 Guidelines for Restarting HRT Study Pills for a Participant Who Has Had a MI or Stroke

CC staff should not actively encourage participants to restart study pills after a new diagnosis of TIA, MI, stroke, or other cardiovascular condition that can increase a participant's risk for a thrombotic event. If the participant wishes to restart (or continue) study pills, advise her to discuss with her primary care provider the advisability of restarting study pills that may contain estrogen. If the participant prefers, she may ask a representative of the CC to discuss this question with her provider. If the participant still wishes to restart (or continue) study pills, and the CC PI approves, the participant may resume her pills.

5.6 Unblinding (Required)

All CC personnel and participants will be blinded to individual participant HRT treatment assignments. All efforts will be made to prevent unblinding of participants for the duration of the trial. However, in some instances of unexpected or abnormal bleeding, it may be necessary for a CC consulting gynecologist or a private primary care provider to be unblinded to ensure maximal participant safety.

Each CC must identify an unblinding officer who will have access to the database function for unblinding. This is usually the CC Data Coordinator. Only the CC consulting gynecologist may be unblinded through the unblinding officer. The unblinding officer may not be involved in clinical activities or the adjudication of outcomes. Unblinding information is restricted to the CC consulting gynecologist and the unblinding officer. The CC consulting gynecologist, however, should be the only person who keeps a record of the participant's treatment arm. Any other documents (e.g., log or participant progress notes) should only include the fact that an unblinding occurred. As long as the unblinded information is limited to these individuals and these persons are not involved in outcome adjudication, the potential for biasing study outcomes is minimal. Bias can further be minimized by maintaining participant blinding, even when unblinding of the gynecologist becomes necessary. Serious complications such as those requiring surgery may necessitate unblinding a small number of participants.

Depending on the clinical findings, unblinding will be considered under circumstances involving either participant safety or management of adverse effects. Such conditions are discussed in *Section 5.4.3 – Management of Vaginal Bleeding.* Should unblinding become necessary, the unblinding officer will execute the database function, which will require input of data (from the CP or CC consulting gynecologist) regarding rationale for unblinding. (See *Vol. 5 - Data System, Section 6.3 – Unblinding Procedures.*)

5.7 Follow-Up Endometrial Biopsies

HRT participants with a uterus may have a follow-up endometrial biopsy to investigate reasons for bleeding while on study pills, or because they have been identified as a participant in the 6% endometrial aspiration subsample done in years 3, 6, and 9.

WHILMA report *Members in Subsample* (*WHIP 1410*) will identify participants in the endometrial aspiration subsample eighteen months before the biopsy is required. CCs should inform participants of the need for this task as soon as the participant is identified. All attempts should be made to obtain a biopsy sample. If entry is not possible, or the participant refuses, obtain a transvaginal uterine ultrasound instead. If the participant refuses the ultrasound, there is no effect on her status in the HRT.

Follow-up biopsies should be sent to local pathologists for evaluation (see *Section. 5.7.1 – Reading of Follow-Up Biopsies*). If abnormal results are identified in either the follow-up of abnormal bleeding or in the subsample pathology report, follow the treatment guidelines in *Section 5.4.3.2—Management According to Endometrial Histology.*

5.7.1 Reading of Follow-Up Endometrial Biopsies

Thorough education and establishment of lines of communication with CC staff (including the CC consulting gynecologists), primary care providers and local pathologists will assure effective, appropriate, and timely care of WHI participants. Send your local pathologist the following WHI Endometrial Histology Classification to aid in their reporting of results. Classifications include:

- No endometrial tissue identified
- Insufficient specimen
- Normal atrophic endometrium
- Normal secretory endometrium
- Normal proliferative endometrium
- Cystic (simple) hyperplasia present
- Cystic (simple) hyperplasia with atypia
- Adenomatous (complex) hyperplasia present
- Adenomatous (complex) hyperplasia with atypia
- Atypia present (carcinoma in-situ [CIS])
- Cancer present
- Other (include description)

The clinical management of women will be based on local pathology readings. There are some general principles that guide management decisions:

- The randomized woman is first a patient and second a participant in the WHI.
- The randomized woman's primary care provider must have the latitude to act "in the best interest" of the participant.

The local pathologist's reading will be sent to the participant's respective CC, which if necessary, will then forward the readings to the primary care provider in a timely fashion.

Section 5
Hormone Replacement Trial

Table of Contents

Contents **Page**

INTRODUCTION 5-1

5.1 HRT Eligibility Issues 5-2

5.1.1 Informed Consent 5-2
5.1.2 Eligibility Based on Baseline Gynecologic Evaluations 5-2
Exclusions Based on Baseline Clinical Breast Exam Findings. 5-2
Exclusions Based on Baseline Pelvic Exam Findings 5-2
Exclusions Based on Baseline Pap Smear 5-2
Exclusions Based On Baseline Endometrial Evaluation 5-3
Exclusions Based on Baseline Transvaginal Uterine Ultrasound 5-4
5.1.3 HRT ”Washout” Period for Screenees Already on HRT 5-5

5.2 Initiating the HRT Intervention 5-6

5.2.1 Step 1 - Introducing the HRT Intervention to the Participant 5-6
Providing the *HRT Handbook* 5-6
Reviewing the Importance of the HRT 5-6
5.2.2 Step 2 - Taking the HRT Study Pills 5-6
Instructions for Taking Pills 5-6
Using the Pill Organizer 5-6
Designing a Reminder System 5-6
Identifying and Building Skills 5-7
Review 5-7
5.2.3 Step 3 - Understanding Symptoms 5-7
Identify Fears and Beliefs 5-7
Reviewing Possible Symptoms 5-7
Review 5-7
5.2.4 Step 4 - Discussion of Routine Monitoring 5-7
Reasons for Monitoring 5-7
Self-Monitoring 5-7
Symptom Monitoring 5-8
Clinical Monitoring 5-8
Review 5-8
5.2.5 Randomization Visit 5-8
Step 1 - Weighing Pills 5-8
Step 2 - Monitoring Adherence and Symptoms 5-8
Step 3 - Reviewing Key Points 5-9

5.3 Follow-Up Contacts with HRT Participants 5-10

5.3.1 Identifying Problems at Participant Contacts 5-10
5.3.2 SERMs Handout 5-10

5.4 Managing Symptoms 5-11

5.4.1 Minor Symptoms 5-11
Educate the Participant About Possible Symptoms 5-11

Participants' Reporting of Minor Symptoms 5-11
Initial Management of Minor Symptoms 5-11
Step-Down Dose Management for Refractory Symptoms 5-13
5.4.2 Management of Menopausal Symptoms 5-14
5.4.3 Management of Vaginal Bleeding 5-16
When to Biopsy 5-18
Management According to Endometrial Histology 5-19
Management According to TVUS Results 5-21
Additional Guidelines for Managing Bleeding after First Year of HRT Study Pills 5-21
Indefinite Use of Additional CEE 0.3 mg Daily. 5-22
Use of Cyclic CEE/MPA for HRT Participants 5-22

5.5 Problems That May Affect Continuation of Study Pills 5-24

5.5.1 Management of HRT After Hysterectomy for Non-Cancerous Condition 5-24
5.5.2 Health Problems Requiring Serious Review 5-24
5.5.3 Health Problems That Require Temporary Discontinuation 5-25
5.5.4 Health Problems Requiring HRT Termination 5-25
5.5.5 Other Issues Requiring Discontinuation of HRT Study Pills 5-26
5.5.6 Guidelines for Restarting a HRT Participant Who Discontinued Pills for 12 Months or More 5-26
5.5.7 Guidelines for Restarting HRT Study Pills for a Participant Who Has Had a MI or Stroke 5-27

5.6 Unblinding (Required) 5-28

5.7 Follow-Up Endometrial Biopsies 5-29

5.7.1 Reading of Follow-Up Endometrial Biopsies 5-29

Figures

5.1 Baseline Endometrial Aspiration Actions 5-5
5.2 Hormone Step-Down Management for Symptoms 5-15
5.3 Continued Menopausal Symptoms Management 5-16
